# BRET and Time-resolved FRET strategy to study GPCR oligomerization: from cell lines toward native tissues

**DOI:** 10.3389/fendo.2012.00092

**Published:** 2012-07-23

**Authors:** Martin Cottet, Orestis Faklaris, Damien Maurel, Pauline Scholler, Etienne Doumazane, Eric Trinquet, Jean-Philippe Pin, Thierry Durroux

**Affiliations:** ^1^Institut de Génomique Fonctionnelle CNRS, UMR 5203,Montpellier, France; ^2^INSERM, U.661, Montpellier and Université Montpellier 1,2,Montpellier, France; ^3^Cisbio Bioassays, Codolet, France

**Keywords:** G protein-coupled receptor, fluorescence, FRET, time-resolved FRET, BRET, fluorescent ligand, oligomer

## Abstract

The concept of oligomerization of G protein-coupled receptor (GPCR) opens new perspectives regarding physiological function regulation. The capacity of one GPCR to modify its binding and coupling properties by interacting with a second one can be at the origin of regulations unsuspected two decades ago. Although the concept is interesting, its validation at a physiological level is challenging and probably explains why receptor oligomerization is still controversial. Demonstrating direct interactions between two proteins is not trivial since few techniques present a spatial resolution allowing this precision. Resonance energy transfer (RET) strategies are actually the most convenient ones. During the last two decades, bioluminescent resonance energy transfer and time-resolved fluorescence resonance energy transfer (TR-FRET) have been widely used since they exhibit high signal-to-noise ratio. Most of the experiments based on GPCR labeling have been performed in cell lines and it has been shown that all GPCRs have the propensity to form homo- or hetero-oligomers. However, whether these data can be extrapolated to GPCRs expressed in native tissues and explain receptor functioning in real life, remains an open question. Native tissues impose different constraints since GPCR sequences cannot be modified. Recently, a fluorescent ligand-based GPCR labeling strategy combined to a TR-FRET approach has been successfully used to prove the existence of GPCR oligomerization in native tissues. Although the RET-based strategies are generally quite simple to implement, precautions have to be taken before concluding to the absence or the existence of specific interactions between receptors. For example, one should exclude the possibility of collision of receptors diffusing throughout the membrane leading to a specific FRET signal. The advantages and the limits of different approaches will be reviewed and the consequent perspectives discussed.

## INTRODUCTION

The analysis of the molecular mechanisms underlying cellular processes reveals the existence of very complicated molecular networks. Each of them is likely to constitute a platform to integrate information. Membrane proteins such as G protein-coupled receptors (GPCRs) are probably one of the first molecular integrators upon cell stimulation. They lead to the activation of one or various signaling pathways depending on the binding of full or biased agonists. Indeed, GPCRs interact with G proteins and/or proteins such as β-arrestins. Their integrating capacities are even larger than expected since, during the last two decades, GPCRs like other membrane proteins such as tyrosine kinase receptors or ionic channels, have been shown to have the propensity to oligomerize (Salahpour et al., 2000; Terrillon and Bouvier, 2004; Milligan, 2010; Lohse et al., 2012).

The emergence of the GPCR oligomerization concept is challenging at different levels and consequently remains a controversial issue. The first difficulty regards molecular and mechanistic aspects. The ability of one GPCR to interact with identical or different GPCRs to form respectively homomers or heteromers opens fascinating perspectives in terms of receptor functioning. However, as experiments have been performed on numerous receptors models, no unifying mechanism regarding size of the oligomers, their stability for example, seems to exist.

The second level regards physiology: the concept has essentially been studied on receptors expressed in heterologous expression systems and various parameters (level of expression, expression of chimeric receptors, and localization of receptors) can deeply impact receptor oligomerization. Whether the data can be extrapolated to physiological context is crucial. Moreover the exact role of GPCR oligomers is far from being well understood and the impact of GPCR oligomerization remains to be established in (patho-)physiology.

Various experimental strategies have been used to study GPCR oligomers: binding experiment, biochemical, or biophysical approaches. Among them, those based on resonance energy transfer (RET) are probably the ones which exhibit the best resolution to conclude to direct receptor interactions. Variants of energy transfer strategies have been developed to explore specific aspects of GPCR oligomerization. In a first part, major findings regarding GPCR oligomerization are briefly reviewed and in the following sections, characteristics, advantages and drawbacks of the various RET approaches have been examined.

## GPCRs FORM OLIGOMERS

As mentioned above, the concept of GPCR oligomerization emerged two decades ago. The demonstration of the direct specific interaction between two proteins is far from being trivial. Various pharmacological- and biochemical-based data have been brought to support the concept of GPCR oligomerization. First, complex curve profiles illustrating ligand binding on GPCRs have been reported for various receptors and have been interpreted as evidence for the existence of GPCR complexes (Mattera et al., 1985; Wreggett and Wells, 1995; Armstrong and Strange, 2001; Rios et al., 2001; Albizu et al., 2006; Springael et al., 2006; Birdsall, 2010), although other hypotheses can also be considered (Chabre et al., 2009). Second, functional trans-complementations upon chimeric or mutated receptor co-expression have been reported for adrenergic α2 and muscarinic M3 (Maggio et al., 1993), angiotensin 2 AT1 (Monnot et al., 1996), and histamine H1 (Bakker et al., 2004) receptors. Third, co-immunoprecipitation experiments illustrating the existence of GPCR complexes have been reported for many receptors, for example for β2 adrenergic (Hebert et al., 1996), δ opioid (Cvejic and Devi, 1997), and metabotropic glutamate mGlur5 (Romano et al., 1996) receptors. Regarding co-immunoprecipitation data, it has been mentioned that they are dependent on the experimental conditions used, and notably on the nature of the detergent and that the functional unit is the monomer (Chabre and le Maire, 2005). Finally, disruptions of GPCR complexes by sequences corresponding to VI and/or VII transmembrane domains (Hebert et al., 1996; Ng et al., 1996) have been reported. All these data, although not definitive, support the idea that GPCRs can form complexes. However, they cannot prove the existence of direct interaction between receptors. Moreover they raise questions regarding the size, the stability, the regulation, and of course the role of such complexes. Biophysical strategies and more specifically RET approaches have been developed to investigate these questions.

### SIZE OF GPCR COMPLEXES

Size of complexes is variable depending on the GPCR model. Metabotropic glutamate receptors (mGluRs) form dimers, covalent disulfide bridges connecting the extracellular domains of two receptors (Pin et al., 2003) and no higher order oligomers were found (Doumazane et al., 2011). By contrast, GABA_B_ receptors have the propensity to form heterotetramers (Maurel et al., 2008; Comps-Agrar et al., 2011a). Regarding receptors of class A, large complexes are been considered for rhodopsin (Fotiadis et al., 2003; Fotiadis et al., 2006), and α1b receptor (Lopez-Gimenez et al., 2007); receptors tetramers have been reported for dopamine receptors (Guo et al., 2008) and monomers for neurokinin 1 receptor (Meyer et al., 2006). It is noteworthy that experimental conditions and more specifically receptor density, have been suspected to impact receptor oligomerization (Chabre et al., 2009).

### STABILITY OF OLIGOMERS

First only considered as monomer and then as stable oligomers, GPCR monomers and higher order oligomers have now been reported to co-exist. For example, co-existence of monomers and dimers and equilibrium between these two forms has been described for M1 muscarinic (Hern et al., 2010) and *N*-formyl peptide (Kasai et al., 2011) receptors. Transient interactions between β1 receptors have also been described, while stable interactions were observed between β2 receptors (Dorsch et al., 2009). Whether this equilibrium is regulated by ligand binding remains an open question since, depending on the receptor considered, contradictory results were reported: for example, muscarinic M1 receptors dimerize upon antagonist binding (Ilien et al., 2009) or is insensitive to antagonists (Goin and Nathanson, 2006); somatostatin receptor 2 dimer dissociates upon agonists binding (Grant et al., 2004) and absence of ligand effect has been reported on numerous GPCR models (for example, Albizu et al., 2010).

### ROLE OF OLIGOMERS

The role of receptor oligomerization is probably the most crucial question but is nonetheless quite poorly characterized. For few receptors, GPCRs oligomerization has been described as essential for receptor trafficking. It has been well illustrated for the GABA_B_ receptor for which the targeting of GABA_B1_ subunit is possible only if it interacts with GABA_B2_ subunit (Pagano et al., 2001). Homodimerization of β2 adrenergic receptor has also been reported to play a major role in exporting receptor from endoplasmic reticulum to the cell surface (Salahpour et al., 2004). Moreover the selective activation of V1a or V2 receptors when engaged in an heterodimer determines the internalization pattern of the receptor (Terrillon et al., 2004).

Oligomerization also plays key roles in receptor activation. Once again this has been well illustrated by the GABA_B_ obligatory heterodimer model. The GABA_B1_ subunit is responsible for orthosteric ligand binding whereas the GABA_B2_ subunit activates G proteins (Kaupmann et al., 1998; Galvez et al., 1999, 2000; Duthey et al., 2002). The generalization of such a crosstalk to other heteromer models (oligomers of different kinds of receptors) remains to be established. It is noteworthy that heterodimerization can impact the pharmacological profile of both receptor. This has been reported for example for opioid receptors (Jordan and Devi, 1999). In the case of homomers (oligomers of identical receptors), their role is not as clear. A few hypothesis and models have shown the possibilities offered by such complexes (Durroux, 2005; Han et al., 2009; Rovira et al., 2010). However, their relevance *in vivo* still remains to be established as homomer formation could impair GPCR function (White et al., 2007; Arcemisbéhère, 2010; Comps-Agrar et al., 2011a), although the opposite has also been established (Pellissier et al., 2011). Identifying oligomeric complexes and understanding how oligomerization can modify receptor signaling is crucial in pharmacology and drug discovery as it can provide unique targets and new ways to specifically address pathologies (Fribourg et al., 2011).

### EXISTENCE OF OLIGOMERS IN NATIVE TISSUES

Most of the experiments regarding receptor oligomerization have been performed on receptor expressed in cell line and whether the results can be extrapolated to receptors *in vivo* remains to be established. Oligomerization of mGluRs and GABA_B_ receptor has been widely accepted. Regarding class A receptors, oxytocin receptor oligomer has been reported in mammary gland in lactating rate (Albizu et al., 2010). Functional trans-complementation of mutant receptors in the absence of functional wild-type receptors in mice (Rivero-Müller et al., 2010; Vassart, 2010) strongly suggests LH receptor oligomerization *in vivo*. Hetero-oligomerization *in vivo* has also been suspected for various GPCR pairs although direct interactions between receptors were not formally demonstrated (González-Maeso et al., 2008; Albizu et al., 2011).

## PRINCIPLE OF RESONANCE ENERGY TRANSFER

In the 1990s, the most popular experimental approaches to demonstrate receptor oligomerization were Western blot and co-immunoprecipitation assays, although false positive interactions can sometimes be observed. These techniques have proved the participation of both proteins to the same complex but not a direct interaction between two receptors.

Only a very few experimental approaches offer a spatial resolution high enough to conclude to a real interaction. Experiments based on RET principle are probably the most adapted to demonstrate a proximity between two proteins. Indeed, RET, formalized by Theodor Förster in the middle of the 20th century, consists in a non-radiative energy transfer occurring between two partners, one being considered as the donor the other as the acceptor (Förster, 1948), which have to fulfill three conditions. First, donor and acceptor should present energy compatibility, i.e., donor emission spectrum and acceptor excitation spectrum should overlap. Second, the donor and the acceptor should present compatible orientation; the transfer is maximal when the donor and acceptor transition dipole moments are parallel and minimum (equal to 0) when they are perpendicular. Finally, energy transfer can take place only if the two partners are in proximity. The efficiency of the transfer is inversely proportional to the sixth power of the distance.

$$E=\frac{R_{0}^{6}}{R_{0}^{6}+r^{6}}$$

where *R*_0_ is the distance corresponding to 50% energy transfer efficiency. Although *R*_0_ depends on the spectral compatibility of the two species and their alignment, it is generally in the range of 30–60 Å. Therefore, because of the spatial resolution offered by RET strategies, RET signals are often interpreted as resulting from direct interactions between partners. Of note, other techniques such as classic microscopy approach and even high-resolution microscopy do not exhibit such high resolutions; they are usually greater than 250 and 30 nm, respectively, and therefore can only provide evidence of receptor co-localization.

Developing an efficient RET-based assays requires to focus on various aspects. First, obtaining a high signal-to-noise ratio is crucial. Different factors can impact this ratio: (i) the overlap of the excitation and emission spectra of the donor and the acceptor. This results in the need to resort to indirect measures of the actual RET, for example, by correcting the measured signal of possible bleed-through and fluorescence contamination. It requires various mathematical operations (Zheng et al., 2002), resulting in a significant decrease of the signal-to-noise ratio; (ii) autofluorescence of the medium and/or the biological preparation and light scattering by cells or membrane preparation often deeply impact the signal-to-noise ratio.

The second aspect regards the labeling of the protein of interest. Initially, experiments were often performed on purified proteins and labeling was achieved**via chemical approaches. Performing similar experiments in a cellular context required novel labeling strategies. This has often been carried out by molecular engineering strategies, i.e., by fusing fluorescent proteins to the protein of interest. Various mutants of the natural green fluorescent protein (GFP) or other fluorescent proteins have been engineered and exhibit fluorescence at various wavelengths.

**FIGURE 1:**
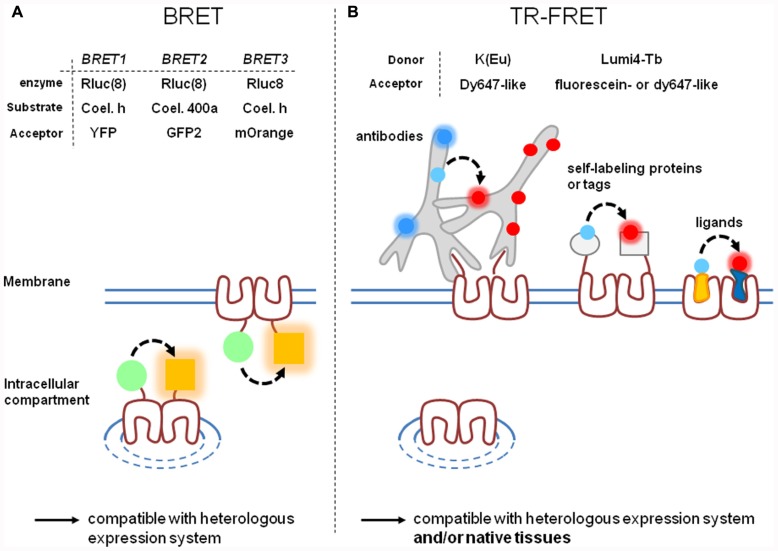
**Comparison of BRET and time-resolved FRET approaches.** BRET **(A)** and TR-FRET **(B)** techniques are actually the most widely used RET techniques since they offer high signal-to-noise ratio. However, they present different characteristics: labeling strategies are often simpler with BRET than with TR-FRET. **(A)** Three variants of BRET depending on the substrate/enzyme complex donor (green circle) and on the acceptor (orange square) have been developed. BRET gives the opportunity to label intracellular or cell-surface targeted receptors. Variants of TR-FRET depending on the fluorescent carrier have been developed. **(B)** TR-FRET opens the possibility to discriminate receptor targeted to the cell surface from those trapped in intracellular compartments. TR-FRET is also more adaptable to different cellular contexts and is the only one to be compatible with receptors expressed in a native context. [Rluc(8), Rluc or Rluc8; Coel. h, Coelenterazine h; Coel. 400a, Coelenterazine 400a (also known as DeepblueC)].

As a solution to these issues, two major strategies have been developed in the last decade: bioluminescent resonance energy transfer (BRET; **Figure 1A**) and time-resolved fluorescence resonance energy transfer (TR-FRET; **Figure 1B**). Interestingly, the use of these two techniques goes beyond the strict GPCR dimerization framework and many aspects of the GPCR life cycle can be analyzed with these approaches.

## BRET STRATEGY

Briefly, BRET is based on the use of a bioluminescent protein, commonly luciferase from *Renilla reniformis* (Rluc), as donor. Therefore, RET occurs without light excitation of the sample leading to a very low background signal, the excitation being chemically triggered (**Figure 1A**). BRET has been optimized along the last two decades and its different implementations (**Figure 1A**) have been recently reviewed (**Ayoub and Pfleger, 2010**). Indeed, Coelenterazine h was first used as substrate of Rluc and yellow fluorescent protein (YFP) as acceptor. Because of the overlap of the donor and acceptor spectra, a second version of BRET (BRET2) has been developed with Coelenterazine 400a (also known as DeepblueC) as substrate for Rluc and GFP as acceptor and displays a better spectral resolution. However, it also exhibits rapid decay kinetics of the substrate and a weak sensitivity because of a low quantum yield when using Rluc (Hamdan et al., 2005; Pfleger et al., 2006). More recently, eight mutations were introduced in the native Rluc to give Rluc8 which shows a fourfold increase in light output (Loening et al., 2006). It can be used in combination either with GFP2 (with Coelenterazine 400a as substrate) or with YFP or a mutant red fluorescent protein (mOrange; Bacart et al., 2008; De et al., 2009; with Coelenterazine as substrate), offering various possibilities to perform BRET with the same donor.

The development of BRET strategy, widely used to characterize receptor interactions, has played a major role in the evolution of the GPCR oligomerization concept (Achour et al., 2011). Interestingly, BRET has also been convenient to show that some receptors such as vasopressin and oxytocin receptors (Terrillon et al., 2003) assemble in oligomers early during their synthesis in the endoplasmic reticulum. Additionally, a combination of bioluminescence and fluorescence complementation and RET strategies have been used to demonstrate that at least four dopamine D2 receptors are located in close molecular proximity in living mammalian cells, consistent with D2 receptor tetramerization (Guo et al., 2008).

On a different note, BRET assays have also been developed to detect various signaling pathway activations. These assays are based on the occurrence of protein interactions consecutive to receptor activation such as G protein or β-arrestin recruitment. Moreover, as mentioned above, the different mutants of Rluc can be associated to various acceptors allowing multiplexing of multicolor BRET. This opens the path for concomitant monitoring of various independent biological processes in living cells (Breton et al., 2010). Lastly, BRET methods present the advantage of being compatible with kinetics measurements since signals can be recorded for up 30 min.

Despite good signal-to-noise ratio and the simplicity to label receptors (Rluc or fluorescent proteins are generally fused to receptor C-terminus), BRET strategies suffer of at least two main drawbacks. First, BRET signals do not discriminate between receptors targeted to the cell surface from those retained inside the cell (**Figure 1A**). Therefore, the BRET signal reflects the behavior of all mature (targeted to the cell surface or internalized) and non-mature receptors. Second, all BRET experiments are based on chimeric receptors expressed in heterologous expression systems. Receptor over-expression and mis-targeting can potentially impact the relevance of the results, especially when BRET is used to prove receptor heterodimerization. Therefore, BRET is not adapted to study receptors expressed in their native context except by using knock-in strategies.

## TIME-RESOLVED FRET STRATEGY

Time-resolved FRET is another relevant RET method to study GPCR oligomerization. It is based on receptors labeled with lanthanides and more specifically with terbium and europium. Lanthanides exhibit long-lasting light emission because of electronic dipole transitions that are formally forbidden. Therefore this photoluminescence is strictly speaking not fluorescence nor phosphorescence since it does not involve singlet-to-singlet or triplet-to-singlet transition (Selvin, 2002). For this reason it should be called lanthanide resonance energy transfer. However, the variations of RET signal in function of the distance between donor and acceptor with lanthanides is similar to those with classic fluorophores, the reason why it has been assimilated to FRET.

**FIGURE 2:**
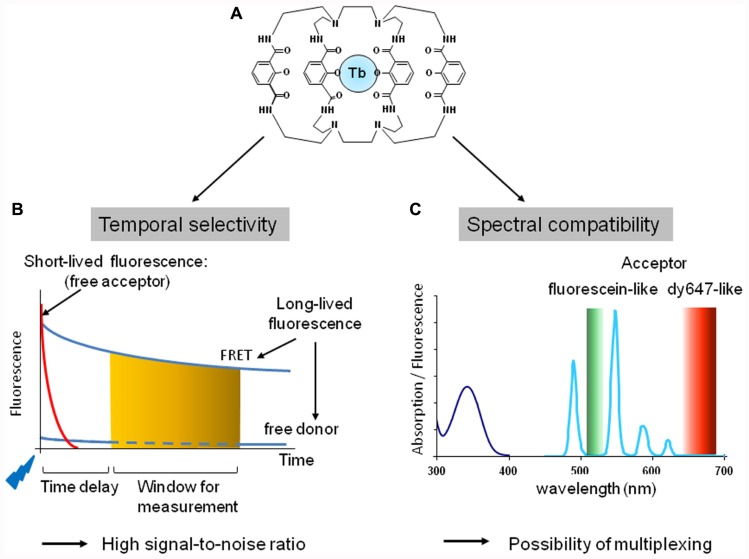
**Fluorescent properties of cryptate of terbium, Lumi4-Tb.** **(A)** Structure of the terbium cryptate (lumi4-Tb). **(B)** Temporal selectivity. The introduction of a time delay (usually about 50 μs) between a flash excitation (blue flag) and the measurement of the fluorescence (orange zone) at the acceptor emission wavelength allows to discriminate long lived from short-lived fluorescence and to increase signal-to-noise ratio. **(C)** Spectral compatibility. Absorption (dark blue line) and fluorescence emission (light blue line) of Lumi4-Tb. The lanthanide cryptate exhibits four emission peaks: 490, 548, 587, and 621 nm. Lumi4-Tb as donor is therefore compatible with fluorescein-like (green zone) and Cy5- or dy647-like (red zone) acceptor to perform TR-FRET experiments.

Two types of cages have been developed to complex lanthanides and enable the labeling of the receptor of interest: (i) chelates display high affinity for europium and terbium ions but the complexation is reversible and can be impacted by the presence of other ions such as Mn^2+^, Mg^2+^, or Ca^2+^; (ii) cryptates, by contrast, offer a greater stability since terbium and europium cannot be released after complexation. An example of structure of cryptate, Terbium cryptate (Lumi4-Tb), is illustrated in **Figure 2A**. Importantly, chelates and cryptates are not just lanthanide carriers but play two other roles. First, they influence the lanthanide fluorescence properties. Indeed they play the role of an antenna since they absorb light and transfer the energy to the lanthanide. This is essential since lanthanides exhibit very weak absorbance (10^4^-fold lower than a classic fluorophore; Selvin, 2002). Moreover the nature of this cage can also impact the emission spectra of the complex. Second, the cage protects lanthanides from quenching by water molecules (Selvin, 2002).

### TIME-RESOLVED FRET EXHIBIT HIGH SIGNAL-TO-NOISE RATIO

The high signal-to-noise ratio provided by TR-FRET strategy is due to various parameters (Mathis, 1995; Bazin et al., 2002).

#### Temporal selectivity

Upon excitation, lanthanide fluorescence half time is in the range of 1 ms while it is in the range of few nanoseconds for classic fluorophores. TR-FRET takes advantage of this property: the introduction of a time delay (typically around 50 μs) between the excitation and the fluorescence signal detection allows discriminating between short-lived and longer-lasting fluorescence. Therefore all short-lived fluorescence provided by the medium, the biological preparation or the direct excitation of the acceptor will be eliminated by the time delay. Only the long-lived fluorescence resulting from the donor or the acceptor engaged in a FRET process will be measured after the time delay (**Figure 2B**).

#### Spectral compatibility

Both europium and terbium cryptates are excited at 300–350 nm. They both exhibit an important Stoke shift and complex emission spectra with multiple fluorescent peaks. For example, europium cryptate trisbipyridine [TBP(Eu)] exhibits four major fluorescent peaks at 585, 605, 620, and 700 nm, while the europium pyridine bisbipyridine (Eu-PBP) has two major peaks at 595 and 615 nm, and two minor peaks at 680 and 705 nm. Terbium cryptate (Lumi4-Tb; Xu et al., 2011) also displays four emission peaks around 490, 550, 585, and 620 nm (**Figure 2C**). This makes europium and terbium cryptates compatible with deep red Cy5- or dy647-like fluorophores to perform FRET. Moreover because of the emission peak around 490 nm, terbium-cryptate is also compatible with fluorescein-like fluorophore as acceptor. By contrast to FRET or BRET strategies based on CFP/YFP or Luciferase/YFP pairs, respectively, europium and terbium cryptate fluorescence are particularly low at the acceptor emission wavelength leading to a reduced bleed through and thus a high signal-to-noise ratio.

#### Orientation dependence

By contrast to BRET or FRET performed with classic fluorophores, the dependence of TR-FRET to the relative orientation of the fluorophore is very weak because the lanthanide emission is not polarized. The relative orientation of the acceptor cannot impact the *R*_0_ more than 12% due to the random orientation of the lanthanide cryptate donor (Selvin, 2002).

### LABELING OF PROTEIN OF THE INTEREST

A second aspect to consider is the method used to label receptors of interest. Depending on the method, labeling can be complete or not, covalent or not, compatible with homogeneous condition or not, bulky or not. All these parameters can have a direct impact on the efficiency of RET and on the detected signal-to-noise ratio (**Figure 1B**).

#### Non-covalent labeling of chimeric receptor with fluorescent antibodies

Early TR-FRET-based strategies consisted in incubating cells expressing receptors of interest with primary fluorescent antibodies conjugated either to lanthanide cryptates or to classic fluorophores (as donors and acceptors, respectively). Specific antibodies for GPCRs with high affinity are difficult to obtain, so antibodies against epitotes such as hemagglutinine, FLAG, 6-Histine, or cMyc, fused to the N-terminus of the receptor have generally been used.

This method has been successfully used to monitor δ-opioid homomers using cMyc- and FLAG-tagged receptors (McVey et al., 2001) indicating the presence of the complex at the cell surface. By contrast, no cMyc-δ-opioid receptor/FLAG-β2-adrenoreceptor-GFP heteromer can be detected using the same approach, despite the presence of the receptors at the cell surface. However, co-expression of δ-opioid receptor-eYFP and β2-adrenoreceptor-*Renilla* luciferase construct resulted in a small BRET signal upon addition of Coelenterazine. This result has been interpreted as the existence of intracellular heteromer complex which are not targeting to the cell surface, illustrating the importance of discriminating cell surface targeted complexes from those retained inside cells.

Since this study, similar results have showed dimerization for numerous receptors targeted at the cell surface: α1A and α1B-adrenergic (Carrillo et al., 2004; Ramsay et al., 2004), CXCR1 and CXCR2 (Wilson et al., 2005), histamine H1 and H4 (Bakker et al., 2004; van Rijn et al., 2006), vasopressin V1a and V1b (Albizu et al., 2006; Orcel et al., 2009), and various types of mGluRs (Kniazeff et al., 2004; Goudet et al., 2005; Hlavackova et al., 2005; Rondard et al., 2006; Brock et al., 2007).

It is noteworthy that two protocols can be used to analyze receptor homodimerization. On the one hand, identical receptors may be fused to two different tags to label each with a specific antibody conjugated either with the donor or the acceptor. The relative expression of one receptor to the other has to be optimized. On the other hand, the receptors may be fused to a single tag and then labeled statistically with a mix of antibodies conjugated either to the donor or the acceptor. In this last condition one should determine the labeling kinetics and concentration to use for each antibody to get a balanced labeling.

Using this antibody-based approach on differentially tagged receptors, several studies have validated the existence of heteromeric complexes, including the GABA_B1_–GABA_B2_ (Maurel et al., 2004), alpha2A–adenosine A1 (Ciruela et al., 2006), and CXCR1–CXCR2 (Wilson et al., 2005) heteromers.

***Advantages and drawbacks***. The antibody strategy to label receptors presents strong and weak points. First, tags fused to the receptors are generally small (6–12 or 15 residues), therefore their impact on the overall conformation of the receptor is generally low, especially if placed at the N-terminus of the receptor. Moreover antibodies available for classic tags such as 6Histidine, FLAG, hemagglutinine, cMyc usually keep good affinities for the tags when fused to the N-terminus. Antibodies when exhibiting high affinities can be used at concentrations lower than 10 nM. This allows carrying out experiments in homogeneous conditions, i.e., without separating the antibody free fraction (not bound onto the tagged receptor) from the bound fraction. Experiments are thus simpler to perform.

Second, antibodies are large and not permeant molecules. Therefore their binding is only possible on cell surface receptors allowing discrimination of cell surface targeted receptor. However, it has recently been shown that similar TR-FRET experiments can also be performed on mildly permeabilized cells expressing C-terminus tagged receptors (Ayoub et al., 2010). The size of the antibodies can also be considered as a weak point since they generate important steric hindrance in the vicinity of the receptors. This can potentially be prejudicial for the binding of at least two antibodies, especially on class A receptors which usually display shorter N-terminus than class B and C GPCRs. Moreover, because antibodies are approximately three times larger than GPCRs, FRET signal between antibodies have to be cautiously interpreted as receptor oligomerization.

Finally antibodies can carry several fluorophores. This has often been considered as an advantage since it increases the fluorescence intensity of GPCR labeling either with donor or acceptor antibodies. However, it does not necessarily increase the signal-to-noise ratio. Moreover, since labeling of antibodies with donor or acceptor fluorophores are usually random, no optimization of the position of fluorophores on antibodies is possible.

Finally, remarks must be made concerning the binding of antibodies to tagged receptor. First, labeling of receptors by antibodies is reversible and time to reach the binding equilibrium can be long depending on receptors. For example, it can exceed 4 h at 4^°^C for HA tagged GABA_B_ receptor (Maurel et al., 2004). Second, saturation of receptor labeling with antibodies can require high concentration, not compatible with homogeneous conditions. Third, ligand binding onto their cognate receptor can modify receptor conformation and therefore impact the access of antibodies to their epitope. Therefore variations of FRET could not reflect variation in the dimerization process but rather a modification of the affinity of antibodies for their epitope. Finally, antibodies are bivalent proteins, and although it has not been reported yet, one cannot exclude that they may artificially drive dimerization of non-interacting receptors (Maurel et al., 2008).

To conclude, TR-FRET strategies with fluorescent antibodies are interesting approaches exhibiting good signal-to-noise ratio. They allow the specific study of receptors targeted to the cell surface. However, their size and their non-covalent binding to receptors undoubtedly constitute a limitation to their use.

#### Covalent labeling of chimeric receptors

Various strategies developed during the past 10 years are based on the fusion of receptors to various peptides, either a self-labeling protein (SLP; also improperly called suicide enzyme) or a sequence recognized by enzymes (**Figure 1B**). TR-FRET experiments have also been performed on purified mutated receptors in which a reactive cysteinyl residue has been introduced.

**FIGURE 3:**
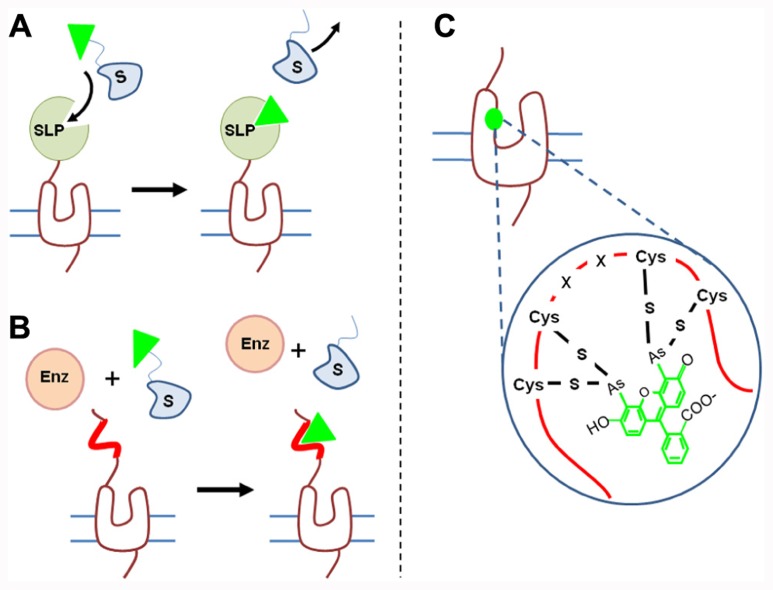
**Strategies to covalently label GPCRs.** **(A)** SLP generally fused to the N-terminus of GPCRs catalyze the transfer of one fluorescent group (green triangle) from the substrate to itself. Various self-labeling proteins such as SNAP-tag, CLIP-tag, or HaloTag have been used to analyze receptor oligomerization. **(B)** Enzyme-based labeling: cells expressing tagged receptors are incubated in the presence of an enzyme such as AcpS and fluorescent substrate. The enzyme (Enz) catalyzes the transfer of one fluorescent group (green triangle) from the substrate to a specific tag incorporated into the receptor sequence (red line). **(C)** FlASH and ReASH strategies consists in introducing into the GPCR sequence a tetracysteine sequence (-C-C-X-X-C-C-) which reacts with fluorescent arsenical derivatives.

***Fusion of receptor to a self-labeling protein***. Several approaches consist in fusing a SLP to the N-terminus of the receptor and providing fluorescent substrates. SLPs can catalyze the transfer of a fluorescent group from the substrate onto itself. For example, the SNAP-tag protein (23 kDa, i.e., two-thirds of GFP), derived from the DNA repairing enzyme O6-alkylguanine-DNA alkyltransferase (or AGT) transfers the fluorescent benzyl group from a fluorescent benzyl guanine substrate to label itself (Juillerat et al., 2003, 2005; Keppler et al., 2003, 2004; Gronemeyer et al., 2006; **Figure 3A**). Mutations have been introduced in the native protein to reduce its size, increase its reactivity, and decrease its ability to bind DNA. Nevertheless, in permeabilized conditions, labeling of the native protein cannot be excluded and that might slightly increase background emission (Gronemeyer et al., 2005; Juillerat et al., 2005).

The efficiency of these strategies has been validated since 100% of the receptor is labeled with the fluorophore (Maurel et al., 2008; Comps-Agrar et al., 2011b). It is noteworthy that until now lanthanide derived fluorescent substrates are not permeant, therefore only receptors targeted to the cell surface, presenting an extracellular SNAP-tag will be labeled. By contrast other substrates such as tetramethyl rhodamine derivatives are permeant, allowing intracellular protein labeling (Gautier et al., 2009). Other SLP-tags have been developed [e.g., CLIP-tag with benzyl cytosine (Gautier et al., 2008), HaloTag (33 kDa) with HaloTag ligands (Zhang et al., 2006)] allowing the labeling of different receptors with reduced cross-reactivity. As mentioned above, these tags are generally fused to the N-terminus of the receptors since their fusion in the receptor extracellular loops generally induces greater conformational modifications.

These strategies have all been used to investigate GPCR oligomerization in various contexts. Several studies have included in their analysis SNAP- or CLIP-tag labeling on different GPCR models to point out their oligomerization (Maurel et al., 2008; Albizu et al., 2010; Ward et al., 2011). Incubation of cells expressing tagged-receptors in the presence of donor- and acceptor-derived substrates leads to the labeling of receptors with one donor or one acceptor fluorophore. The existence of a TR-FRET signal indicates proximity between receptors and has been interpreted as receptor dimerization.

Moreover, the absence of impact of agonist and/or antagonist binding on receptor oligomerization as reported for vasopressin, oxytocin and dopamine receptors (Albizu et al., 2010), suggest the stability of the interaction between receptors, or at least, that the equilibrium between monomers and oligomers, if any, is not affected by ligand binding. Whether this result could be generalized to other receptors remains to be established but it seems consistent with previous data (Terrillon et al., 2003).

This method has also been used to go one step further and demonstrate that GABA_B_ receptor forms higher order oligomers (Maurel et al., 2008). Significant TR-FRET signals have been recorded between GABA_B1_ and GABA_B2_ subunits and also between two GABA_B1_ subunits if co-expressed with GABA_B2_ subunit. By contrast a weak TR-FRET signal between two GABA_B2_ subunits when expressed with GABA_B1_ subunits has been reported. These results indicate the formation of GABA_B1/B2_ tetramers, GABA_B1_ subunit constituting the interface between the two GABA_B1_/GABA_B2_ dimers (Maurel et al., 2008; Comps-Agrar et al., 2011a). This result is in accordance with previous microscopy studies suggesting oligomeric complex of rhodopsin in native disk membrane (Fotiadis et al., 2003) or with combined bioluminescence/fluorescence complementation and energy transfer (Lopez-Gimenez et al., 2007; Carriba et al., 2008; Guo et al., 2008), suggesting that GPCRs can form larger oligomers.

SNAP- and CLIP-tag labeling have also been associated to study receptor heterodimerization. This strategy has been well exemplified by the analysis performed on mGluRs. Doumazane et al. (2011) have reported the existence of heterodimers between mGluR 1 and 5 on the one hand or between mGluR 2, 4, 7, and 8 on the other hand. No significant TR-FRET signal was observed between receptors of these two groups. Regarding receptors of class A, using the SNAP-tag and CLIP-tag strategy, orexin OX1 and cannabinoid CB1 receptors have been shown to oligomerize and the hetero-complex appears to be more sensitive than orexin homo-oligomers to orexin A regulation (Ward et al., 2011).

***Fusion of receptor to sequence recognized by enzyme***. A second approach consists in introducing a sequence recognized by an enzyme into the receptor of interest. The enzyme will catalyze the transfer of a fluorescent group from a fluorescent substrate to the sequence introduced onto the receptor (**Figure 3B**). For example phosphopantetheinyl transferase (PPTase) called acyl-carrier protein synthase (AcpS) will transfer the phosphopantetheinyl group of coenzyme-A to the acyl-carrier protein (ACP), a 8.7-kDa sequence added to the receptor. Once again, the sequence should be accessible to the enzyme and thus located on the extracellular side (Monnier et al., 2011). Such a sequence is much smaller than SNAP-tag or HaloTag and therefore its insertion into the receptor sequence may be less disturbing.

This strategy has been used in association with the SNAP-tag method to perform GABA_B_ subunits orthogonal labeling and study receptor transactivation (Monnier et al., 2011). It has also been used with classic fluorophores to study class A neurokinin NK1 receptor oligomerization (Meyer et al., 2006).

***Receptor labeling via introduction of reactive cysteinyl residue***. Introduction of fluorophores onto receptors can be achieved by using cysteinyl residue reactivity. Because cysteinyl group are often present in protein sequences, such a labeling can only be performed on purified receptor and not on receptor in their membrane context to avoid a large non-specific labeling. Labeling of receptor with Lumi4-Tb can be achieved by incubating purified receptors with maleimide derivatives (Rahmeh et al., 2012) and receptors can be labeled at one specific position by introducing a cysteine at this position and by mutating all other reactive cysteines. Labeling with acceptor fluorophore can be achieved by using FlAsH and ReAsH methods. It consists in introducing a tetracysteine sequence (two cysteine pairs separated by two amino acid residues, CCXXCC) which exhibits a high affinity for green or red fluorescent arsenical derivatives (Ju et al., 2004; Zürn et al., 2010; **Figure 3C**). Thanks to the combination of these two labeling methods, Rahmeh et al. (2012) have demonstrated conformational modification of vasopressin V2 receptor upon agonist, partial agonist or inverse agonist binding.

*Advantages and drawbacks*. The above covalent labeling strategies offer a wide range of advantages. First, covalent labeling constitutes an interesting alternative to antibodies. It induces a lesser steric hindrance than antibody labeling. As mentioned above this is particularly interesting for class A receptors which generally have N-terminus shorter than class C receptors. Second, various “colors” can be used on the same fused receptor construction by changing the fluorophore linked to the substrate. Receptor homomerization can then be simply studied by incubating cells expressing one receptor with two different fluorescent substrates. Optimization of the labeling requires comparing kinetics of labeling with the different substrates. Third, receptor labeling is irreversible and faster than antibody labeling since 1 h is sufficient to label 100% of the receptors (Maurel et al., 2008). It is noteworthy that more reactive SNAP- and CLIP-tag mutants have recently been developed to get a faster labeling of receptors (Sun et al., 2011). Fourth, TR-FRET methods combined to efficient labeling strategies are convenient to follow receptor conformational modification as shown by Rahmeh et al. (2012) on purified receptors. Conformational changes can also be monitored by SNAP- and CLIP-tag labeling with classic fluorophores to develop sensors of different molecules such as sulfonamides (Brun et al., 2009; Monnier et al., 2011). These techniques have proven to be compatible with cellular assays (Brun et al., 2011). Therefore, the development of a sensor to follow ligand induced receptor conformational modifications or intracellular protein binding is potentially achievable.

These strategies to perform orthogonal labeling require some optimization steps. One essential step is the determination of substrate concentrations to label receptors. Using higher concentrations accelerates the kinetics of receptor labeling but also increase the cross-reactivity of substrates for SLPs (e.g., benzylcytosine presents a cross-reactivity to SNAP-tag). Finally, high concentrations of substrates are not compatible with experiments performed in homogeneous conditions. To get around this problem, one interesting alternative consists in using a substrate conjugated to both a fluorophore and a quencher. The probe becomes highly fluorescent only upon reacting with the SLP (Sun et al., 2011).

Getting positive and negative control to demonstrate receptor oligomerization is an essential point for the validation of this concept. The negative control is probably the most difficult to get. First, FRET can be observed if the expression of the partners is high enough to get random collision of receptor diffusing at the cell surface. Therefore, variation of the receptor expression level can be at the origin of inconsistence between published data. For example, using the same receptor labeling approach, Doumazane et al. (2011) reported the absence of heterodimerization between mGluRs mGluR2 and 5, while a significant signal has been observed by Delille et al. (2012). One important criterion to conclude to the specificity of the interaction is that FRET efficiency should be constant and independent of the level of receptor expression. An alternative control can be to verify the saturation of the FRET signal when the expression of the acceptor is increased and the expression of the donor is kept constant.

Finally, all these strategies, by contrast to BRET, enable the distinction between tagged receptors targeted to the cell surface from those trapped inside cells. On the other hand, they are not applicable to study wild-type receptors expressed in native tissues.

#### Non-covalent labeling of wild-type receptor

As mentioned above, receptor oligomerization potentially opens new perspectives regarding GPCR functioning. However, the concept needs to be validated in a native context and not only on receptors expressed in cell lines. Indeed, various biases could impair the relevance of FRET data obtained in cell lines. The exact impact of using chimeric receptors instead of wild type receptors, of high receptor expression levels, or of different receptor targeting depending on the cell line used, is difficult to evaluate. Thus, the validation of results in a native context is important. This is even more crucial regarding GPCR heteromerization. The demonstration of the existence of heteromers in a cell line can be potentially relevant only if in native tissues receptors are at least expressed at the same time, in the same cell and in the same subcellular compartment. However, native contexts impose constraints since the receptor sequence, the level of expression or the targeting cannot be modified.

Two strategies can be used to label receptors in native tissues. First, antibodies have been considered to fluorescently label endogenous receptors. However, they are large molecules generating steric hindrance and getting specific and high affinity antibodies again GPCRs has proven difficult. These two reasons make antibodies not necessarily the best tools for demonstrating direct receptor interactions. Antibodies produced by Camelids could be a good alternative to conventional antibodies since they are much smaller (17 vs. 150 kDa). Moreover they can recognize different epitopes usually not recognized by conventional antibodies and notably clefts such as ligand binding pockets or enzyme active sites (De Genst et al., 2006; Harmsen and De Haard, 2007). Therefore, besides their small size, they open new perspectives in term of molecular recognition and specificity.

A second strategy based on fluorescent ligands presents several advantages (**Figure 1B**). Ligands are usually smaller molecules, especially regarding GPCRs of class A and C, and can exhibit high affinities for GPCRs. They are therefore potentially suitable to study receptor oligomerization insofar as their fluorescent derivatives maintain high affinities for their cognate receptors. First attempts to demonstrate GPCR oligomerization with fluorescent ligands have been performed on luteinizing hormone and somatostatin receptors (Roess et al., 2000; Patel et al., 2002). However, the sensitivity of the approach was insufficient because of a low signal-to-noise ratio. TR-FRET strategy based on fluorescent ligands represents an interesting alternative method. This has been carried out for peptidic ligands; vasopressin and oxytocin antagonist and agonist derived with lanthanide cryptate as donor (Albizu et al., 2007, 2010) and d2, dy647, and fluorescein (Durroux et al., 1999; Terrillon et al., 2003) as acceptors were synthesized. Surprisingly, this strategy has also been successfully adapted to smaller bioamine ligands. Indeed one could have predicted that adding fluorophores bigger in size than the ligands, such as lanthanide cryptates, should deeply impact the affinity of the latter. The syntheses of lanthanide cryptate labeled derivatives of *N*-(*p*-aminophenethyl) spiperone (NAPS) and (±)-4^′^-amino-2-(*N*-phenethyl-*N*-propyl)-amino-5-hydroxytetralin (PPHT), respectively antagonist and agonist of the dopamine D2 receptor have recently been reported (Albizu et al., 2010). Both ligands exhibit affinities in the 5 nM range for the dopamine D2 receptor. These data are very encouraging since they strongly suggest that development of lanthanide cryptate derived ligands is achievable with a large range of ligands.

TR-FRET strategy based on fluorescent ligands has been validated on V1a and V2 vasopressin receptors, on oxytocin and dopamine receptors with at least five sets of fluorescent ligands (Albizu et al., 2010). It has been shown that TR-FRET signal is not observed on mock cells, abolished in the presence of an excess of unlabeled ligand and that its variation in function of donor/acceptor ratio follows a bell-shaped cure. Therefore these data support that TR-FRET is dependent on the receptor expression and the occupancy of the binding sites with fluorescent ligands, demonstrating the specificity of the TR-FRET signal. It has also been observed that TR-FRET signal obtained with fluorescent agonists is weaker than with fluorescent antagonists. This result has been related to the negative cooperative binding of agonists, in contrast to antagonists, and strongly supports that TR-FRET signal does not result from random collision of receptor diffusing at the cell surface. Indeed when considering the collision hypothesis of monomeric receptors, agonists or antagonists should lead to the same TR-FRET signal for the same level of receptor occupancy.

Similar experiments have been carried out on oxytocin receptors expressed in the mammary gland of lactating rat and consistent results have been obtained proving the existence of oxytocin receptor homodimers in this tissue. Moreover, experiments performed on tissues patches clearly indicate the targeting of receptor dimer to the cell surface. These data validate the fluorescent ligand-based TR-FRET strategy to prove the existence of receptor oligomers in native tissues.

***Advantage and drawbacks***. A large set of fluorescent ligands has been synthesized for numerous GPCRs. Whether the fluorescent derivatives will exhibit high affinity for their cognate receptor remains to be established but the example of D2 dopamine ligands proves that the development of such ligands is feasible. The sensitivity of the technique is dependent on the affinity of the ligand for its receptor. Ligands exhibiting affinity in the nanomolar range are suitable for such experiments since experiments can be carried out in a 96-well plate format and in homogeneous conditions. The absence of washing steps makes the experiments very simple to perform (Cottet et al., 2011) and more reproducible. Moreover, the TR-FRET kinetics can simply be performed and the time to reach equilibrium is easily determined. In the case of low affinity fluorescent ligands or strong negative cooperative binding of one ligand between two binding sites, one could have expected to perform FRET measurements after washing steps. However, dissociation kinetics of ligands can be rapid and therefore incompatible with such a protocol. Finally, because several examples of negative cooperative binding of agonist have been reported (Urizar et al., 2005; Albizu et al., 2006; Springael et al., 2006; Han et al., 2009), it seems more relevant to use fluorescent antagonists to get a double labeling of binding sites within a dimer. It underlines that FRET signal is strongly dependent on cooperative binding mechanisms between ligands.

The approach has been validated on one receptor expressed in a native context, the oxytocin receptor expressed in mammary gland of lactating rat. Indeed this receptor model is interesting since it is highly expressed in this tissue. Whether the method is applicable to tissues expressing receptors at a lower density remains to be established.

Data must be interpreted with caution. Indeed the absence of FRET signal is not necessarily a proof of oligomer absence. It can also be explained by the binding of only one ligand because of a high negative cooperative binding or because of the existence of hetero-oligomers.

## CONCLUSION AND PERSPECTIVES

RET techniques have provided very interesting experimental solutions to study receptor complexes. Indeed, the resolution of RET approaches is <10 nm, far below all conventional optical microscopy techniques. A significant RET signal has thus been interpreted as direct interactions between receptors while conventional microscopy can only conclude to receptor co-localization. Are all RET approaches equivalent to study receptor oligomerization? Certainly not as BRET and TR-FRET are significantly more sensitive with a higher signal-to-noise ratio, and both techniques provide the possibility to perform multiplexing. BRET offers the simplicity of receptor labeling performed by bioengineering techniques. This is a strong advantage but also a disadvantage since it is impossible to distinguish the receptors targeted to the surface or trapped inside the cell. Moreover, although various pairs of donor/acceptor have been developed, all the donors are derived from Rluc. Whether the development of a different and smaller luminescent donor is conceivable remains an open question. TR-FRET displays a larger panel of tools for receptor labeling. Regarding the labeling step, TR-FRET is a little bit more complicated. Antibodies are large molecules inducing a steric hindrance which can be, on some receptor models, prejudicial for observing signals of large amplitude. Covalent labeling techniques offer some advantages with much smaller tags but they generally need additional labeling and washing steps. TR-FRET based on fluorescent ligands is an interesting alternative since, to our knowledge, it is the only technique that can be applied to wild-type receptors expressed in a native context. This constitutes a breakthrough because the validation of the concept of GPCR oligomerization in physiology is crucial.

**FIGURE 4:**
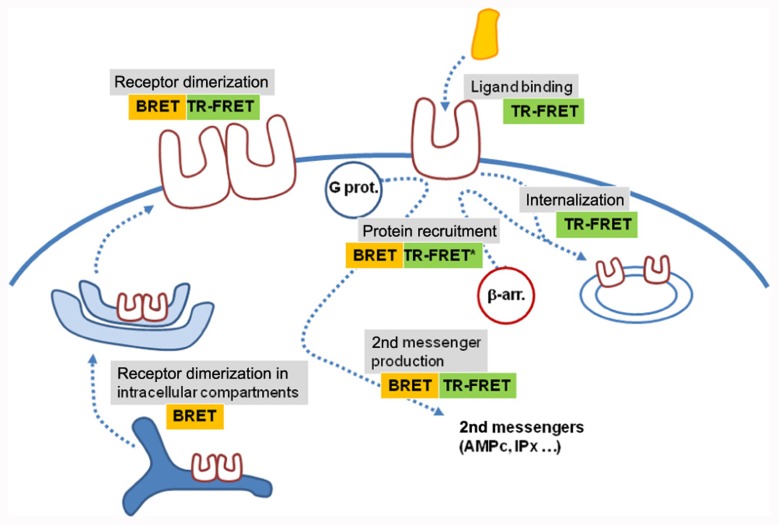
**BRET and time-resolved FRET assays application.** Various BRET and TR-FRET assays have been developed to measure ligand binding, receptor activation through protein recruitment or second messenger production, receptor dimerization or receptor trafficking. Most of them are compatible with high-throughput screening. Recent developments have shown that these techniques are also potentially compatible with high-content screening, opening new perspectives in the use of RET approaches (*means that TR-FRET experiments were performed on mildly permeabilized cells expressing C-terminus tagged receptors).

During the last 20 years, RET techniques became very popular for GPCR oligomerization studies. What are the perspectives for BRET and TR-FRET in the next decade? One major aspect is probably their use in microscopy. Both techniques have been adapted to microscopy constraints (Coulon et al., 2008; Rajapakse et al., 2010). Of note, many assays based on BRET or TR-FRET approaches have been developed in the last decades to measure ligand binding (Ilien et al., 2003; Tahtaoui et al., 2005; Albizu et al., 2007; Zwier et al., 2010), second messenger production (Trinquet et al., 2006), receptor internalization (Zwier et al., 2011), or protein recruitment such as β-arrestins (Angers et al., 2000; **Figure 4**). A number are compatible with high throughput screening (**Figure 4**; Boute et al., 2002). The adaptation of BRET and TR-FRET to microscopy opens new perspectives since both techniques will be compatible with high content screening. Because TR-FRET based on fluorescent ligands is convenient to study receptor oligomerization in native tissues, one can expect that further development of the techniques will allow the study of the role of receptor homomers and heteromers in real life.

Others strategies have recently emerged to investigate GPCR oligomerization. Time-resolved fluorescence anisotropy approach is based on the energy transfer between two identical fluorescent proteins, for example two YFPs. The transfer of energy results in a decrease in the polarization of the fluorescence emission. This approach has recently been used to study 5HT-1A receptor oligomerization (Paila et al., 2011). Other studies have combined high-resolution microscopy and single particle tracking (Hern et al., 2010; Kasai et al., 2011). As such, they open new perspectives since kinetics of GPCR complex dissociation can be monitored. However, high-resolution microscopy techniques on live cells do not yet display a resolution compatible with a definitive identification of GPCR complexes as oligomers. Fast, three-dimensional super-resolution imaging of live cells has recently been described when labeling light chain of clathrin fused to SNAP-tag and a resolution of 30 nm has been reported (Jones et al., 2011). This resolution is thus at least threefold greater than FRET resolution while FRET technique resolution is better than 10 nm. Therefore these techniques offer a complementary point of view to study GPCR oligomerization.

## Conflict of Interest Statement

Etienne Doumazane is employee of Cisbio, which develops HTRF compatible fluorescent products and therefore may gain financially through publication of this paper. Part of the work of CNRS UMR 5203 has been financially supported by Cisbio.

## Abbreviations

BRET: bioluminescent resonance energy transfer
FRET: fluorescence resonance energy transfer
GFP: green fluorescent protein
RET: resonance energy transfer
SLP: self-labeling protein
TR-FRET: time-resolved fluorescence resonance energy transfer
YFP: yellow fluorescent protein
